# TSVdb: a web-tool for TCGA splicing variants analysis

**DOI:** 10.1186/s12864-018-4775-x

**Published:** 2018-05-29

**Authors:** Wenjie Sun, Ting Duan, Panmeng Ye, Kelie Chen, Guanling Zhang, Maode Lai, Honghe Zhang

**Affiliations:** 1 Department of Pathology, Key Laboratory of Disease Proteomics of Zhejiang Province, School of Medicine, Zhejiang University, Hangzhou, 310058 China; 2 Department of Toxicology, School of Medicine, Zhejiang University, Hangzhou, 310058 China; 3 Hikvision Digital Technology, Hangzhou, 310051 China; 4 Department of Integrative Biology and Physiology, University of California, Los Angeles, Los Angeles, CA USA

**Keywords:** Splicing variant, Alternative splicing, TCGA, Cancer, Visualization tools

## Abstract

**Background:**

Collaborative projects such as The Cancer Genome Atlas (TCGA) have generated various -omics and clinical data on cancer. Many computational tools have been developed to facilitate the study of the molecular characterization of tumors using data from the TCGA. Alternative splicing of a gene produces splicing variants, and accumulating evidence has revealed its essential role in cancer-related processes, implying the urgent need to discover tumor-specific isoforms and uncover their potential functions in tumorigenesis.

**Result:**

We developed TSVdb, a web-based tool, to explore alternative splicing based on TCGA samples with 30 clinical variables from 33 tumors. TSVdb has an integrated and well-proportioned interface for visualization of the clinical data, gene expression, usage of exons/junctions and splicing patterns. Researchers can interpret the isoform expression variations between or across clinical subgroups and estimate the relationships between isoforms and patient prognosis. TSVdb is available at http://www.tsvdb.com, and the source code is available at https://github.com/wenjie1991/TSVdb.

**Conclusion:**

TSVdb will inspire oncologists and accelerate isoform-level advances in cancer research.

**Electronic supplementary material:**

The online version of this article (10.1186/s12864-018-4775-x) contains supplementary material, which is available to authorized users.

## Background

During transcription in eukaryotes, alternative splicing (AS) of message precursor RNA generates splicing variants for a single gene, and particular exons may be included or excluded. It was estimated that approximately 92-94% of human genes undergo AS [1]. As one of the most common mechanisms associated with gene regulation [2], AS has emerged as a vital mechanism in tumorigenesis that regulates the function of cancer-related genes [3]. Aberrant splicing patterns are closely related to tumor progression [4]. For example, misregulation of splicing caused by splicing factor Serine And Arginine Rich Splicing Factor 1 (SRSF1) can lead to the malignant transformation of normal mammary cells [5]; we also reported that Serine And Arginine Rich Splicing Factor 6 (SRSF6) promotes tumor progression by regulating AS and might be a potential therapeutic target [6]. Thus, splicing variants could be potential biomarkers [7] and therapeutic targets in cancer studies.

The Cancer Genome Atlas (TCGA) project (http://cancergenome.nih.gov) has incorporated a vast bulk of genomic sequences, epigenetic profiles, transcriptomes and multidimensional clinical datasets. It is an excellent source for exploring and validating genes of interest through the TCGA RNA-Seq.

Information on splicing variants can be identified from RNA Sequencing data through software such as Cufflinks, RSEM (RNA-Seq by Expectation Maximization), Kallisto and MapSplicing [8–10]. Tools such as TCGASpliceSeq [9] and ISOexpresso [11] provide users with alternative splicing patterns and isoform expression data between normal and tumor cells. However, detailed clinical information, such as the tumor grade, race and survival time, are not provided by the existing tools that investigate splicing variants.

To address these questions, we introduced TSVdb, an interactive web-portal, to perform comparative analysis on splicing variants across tumor subgroups using TCGA RNA-Seq datasets from 33 tumors and 30 clinical variables.

TSVdb presents a well-organized visualization of exon/junction usage and splicing patterns, which enable users to readily and quickly access, analyze, and interpret splicing variants for interesting genes. Users can investigate the isoform expression between tumor subgroups and the association of splicing variant expression with overall survival.

We believe that TSVdb provides a user-friendly platform for researchers to maximize TCGA utilization and unearth more potential cancer biomarkers.

## Implementation

### Data collection

TCGA (version 20160128) level 3 data were downloaded from the TCGA FTP site Firehose. The data included the gene, isoform RSEM data, exon, and junction-normalized read count data (UNC illuminaHiSeq_RNASeqV2) and clinical data (Merged_clinical_level_1). Because the data in Firehose no longer updates, data updates will use the TCGA data in the GDC data portal in the future. The current TCGA data version was noted in the footer on the TSVdb plot page. The RNA-Seq data transformation was accomplished with the R software version 3.2.3. Genes with Entrez IDs in both the TCGA data and annotation package *org.Hs.eg.db*(3.2.3) were used. The annotation package *TxDb.Hsapiens.UCSC.hg19.knownGene*(3.2.2) was used to annotate the isoform, exon and junction data in the TCGA datasets. The annotation of the TCGA exon and junction data was performed by overlapping the locations of the exons/junctions with the isoform range. The annotation package was also used to plot the transcript isoform structure.

### Data manipulation and transformationn

The related clinical information for each cancer type was selected and prepared for each tumor. Thirty clinical variables were chosen and processed (Additional file 1: Table S1). A cut-off value was used to make the numerical variables classified variables; if there were too many classes, a combination was applied to reduce the class number. The transformation methods were as follows:

- The number of packs smoked per year smoked, which was an integer variable. The cut-off values were set to 10 and 100 to create the following three categories: (1) “less than 10”, “less than 100”, and “greater than 100” packs smoked per year.
- Overall survival. The day_to_death and days_to_last_follow-up variables were used to generate the time and event variables for overall survival. The event was set to “death” if a patient had a defined non-zero day_to_death value; otherwise, the event variable was considered “censored”. The survival time was set to the larger variable among the day_to_death and days_to_last_follow-up. The survival status was coded as 0 (live or censored) or 1 (death).
- Stage. The class number was reduced to 5 (I, II, III, IV, and X) for the pathology_stage, clinical_stage and masaoka_stage.
- Risk factors of LIHC. “Alpha-1 antitrypsin deficiency”, “hemochromatosis” and “other” were combined into “others”. “Alcohol consumption”, “hepatitis b” and “hepatitis c” were classified by themselves due to their large proportion.
- Alcohol consumption per day was divided into “0” and “ > 0”.
- The number of pregnancies was grouped into six classes, which defined the number of pregnancies as either “1”, “2”, “3”, “4”, “5” or “ > 5”.

“Indeterminate” data were shown as “UNDEFINED”. Additional file 1: Table S1 shows the phenotype statistics for the tumor types.

All the data described above were deposited into the NoSQL database MongoDB (See Additional file 2 for the database scheme).

### Website and Data Visualization

The nodejs scripts were used to provide the private APIs for the web-end. There were three APIs with the following functions: (1) Autocompleting the gene symbol or alias, (2) Validating the symbol or the input Entrez ID, (3) Querying MongoDB and then returning the clinical variable list for the queried tumor, and (4) Querying MongoDB and then returning the data to draw the results. The JavaScript libraries d3.js [12] (version 3.0.6, http://d3js.org/) were used to construct the interactive SVG graph. The d3.js-based interactive KM-plot was modified from Nick Strayer’s code (http://bl.ocks.org/nstrayer/). The box plot and other distribution charts were modified from Andrew Sielen’s code (https://github.com/asielen/D3_Reusable_Charts). The SVG graph download function was adapted from A. Gordon’s solution (https://github.com/agordon/d3export_demo).

1 $$\begin{array}{@{}rcl@{}}  & y_{ij} = \begin{cases} \min \left(\frac{x_{ij}}{e_{j} \cdot Q_{95,i}}, 1\right) & \text{if} \; Q_{95,i} > 0.05 \\ \min \left(\frac{x_{ij}}{e_{j} \cdot 0.05}, 1\right) & \text{if} \; Q_{95,i} \leq 0.05 \end{cases} \end{array} $$

2 $$\begin{array}{@{}rcl@{}}  & e_{j} = \frac{\sum_{i=1}^{n} x_{ij}}{n} \end{array} $$

**Fig. 1:**
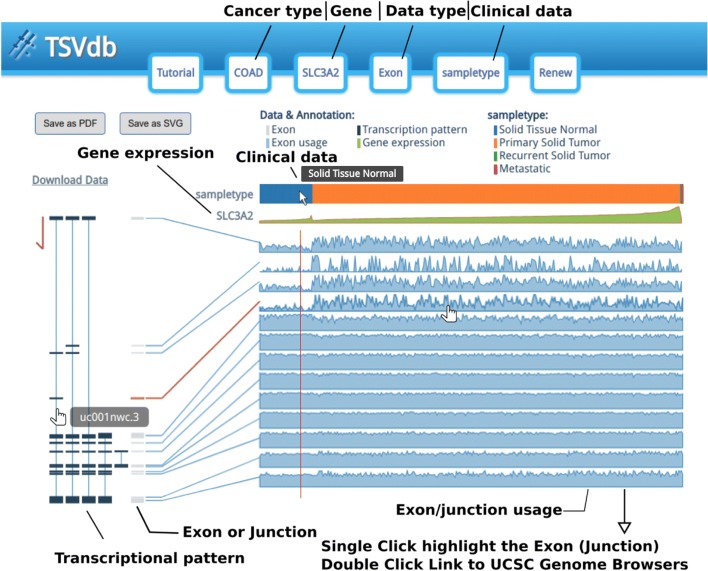
An illustration of the TSVdb database. The top of the web page displays the query parameters buttons; under those buttons were the figure legends. Under the legends were the main results. The sample type, gene expression and exon/junction usage (patients are presented in columns arranged according to their gene expression from low to high in each group and the exons/junctions are arrayed in rows) are displayed on the right side and from the top down. The left side shows the gene transcriptional pattern, in which the thin lines represent the introns and boxes connected by the lines represent the exons corresponding to the rows on the right side. Hovering or clicking on the rows will highlight the corresponding exon in the transcription pattern and double clicking on the rows will open the UCSC Genome Browser in a new tab/window and showing the gene structure. Additionally, clicking on the isoform structure lead to isoform-specific expression data or a survival curve

The exon/junction usage value *y*_*ij*_ displayed in the main results (Fig. 1) was derived from the exon/junction quantification value *x*_*ij*_ with a series of scalings and normalizations Eq. (1). The effects of the normalization are shown in Additional file 1: Figure S2 for a gene that has *n* exon/junction values (*i*=1,…,*n*) in *m* samples (*j*=1,…,*m*). First, following the idea of a “splicing index” [13], each sample’s exon/junction quantification value was divided by the expression quantity *e*_*j*_ of the gene to which the exon belongs Eq. (2). The gene expression quantity was estimated by averaging the quantification of the exons/junctions. Therefore, the gene expression effect was removed and the AS event was highlighted. Next, the d3.js linear scale was used to map the normalized exon/junction values to the graph coordination. The interval of the normalized exon/junction values (domain argument in scale function in *D3*) was set to (0,*Q*_95,*i*_) (95% quantile) to diminish the outlier’s impact, which may minimize the differences in the AS events between groups. Furthermore, if *Q*_95,*i*_<0.05, which indicates the exon/junction expression quantity, is relatively small when corresponding to the gene expression quantity, the upper bound of the interval would be set to 0.05.

**Fig. 2:**
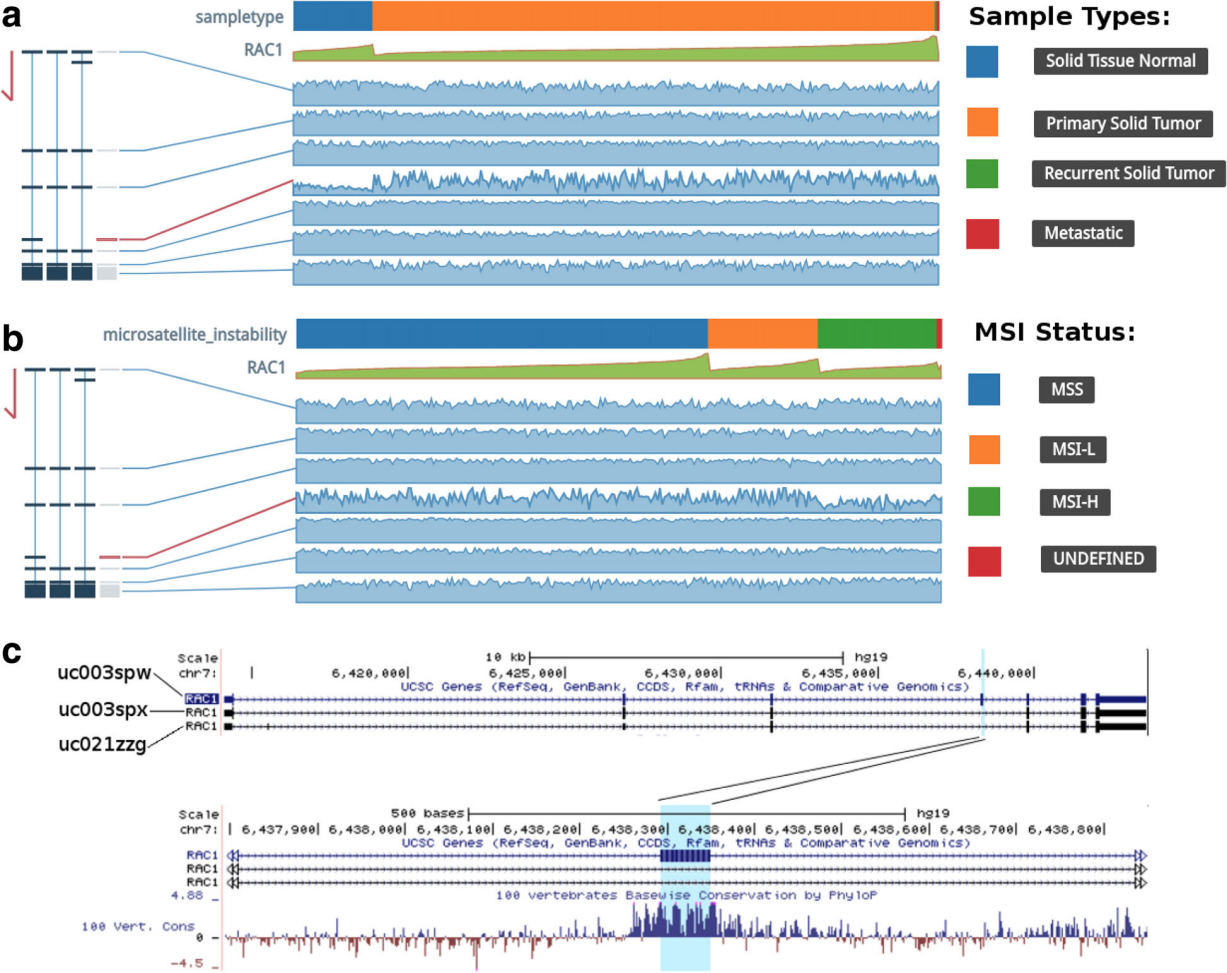
Visualization of the TCGA data for RAC1 in colon adenocarcinoma using TSVdb. **a** The exon usage results in the different RAC1 sample types. **b** The exon usage results for different RAC1 MSI statuses using TSVdb. **c** RAC1 isoforms and annotation of the fourth exon from the UCSC Genome Browser

## Results

### TSVdb use

Four dialogs were initially used to input the tumor type, exon/junction and clinical data for a specific gene. After finishing the input, the main output window showed the clinical information, gene expression, exon/junction usage and isoform structure diagram (Fig. 1). As was shown, the samples were divided into two or more subgroups according to their clinical information, e.g., “Solid Tissue Normal” and “Primary Solid Tumor”. The samples in each group were arrayed by their gene expression levels, which helped to distinguish the correlation between the isoform expression and gene expression. Meanwhile, the shadowed-line charts display the exon/junction usage values for each sample to facilitate the recognition of alternative splicing. Links to the UCSC Genome Browser also offer the exon/junction’s loci as well as further annotation information such as the conservation of the exon sequence, single nucleotide polymorphisms, and mutations, so that researchers can gain a full-scale understanding of the exon or junction.

**Fig. 3:**
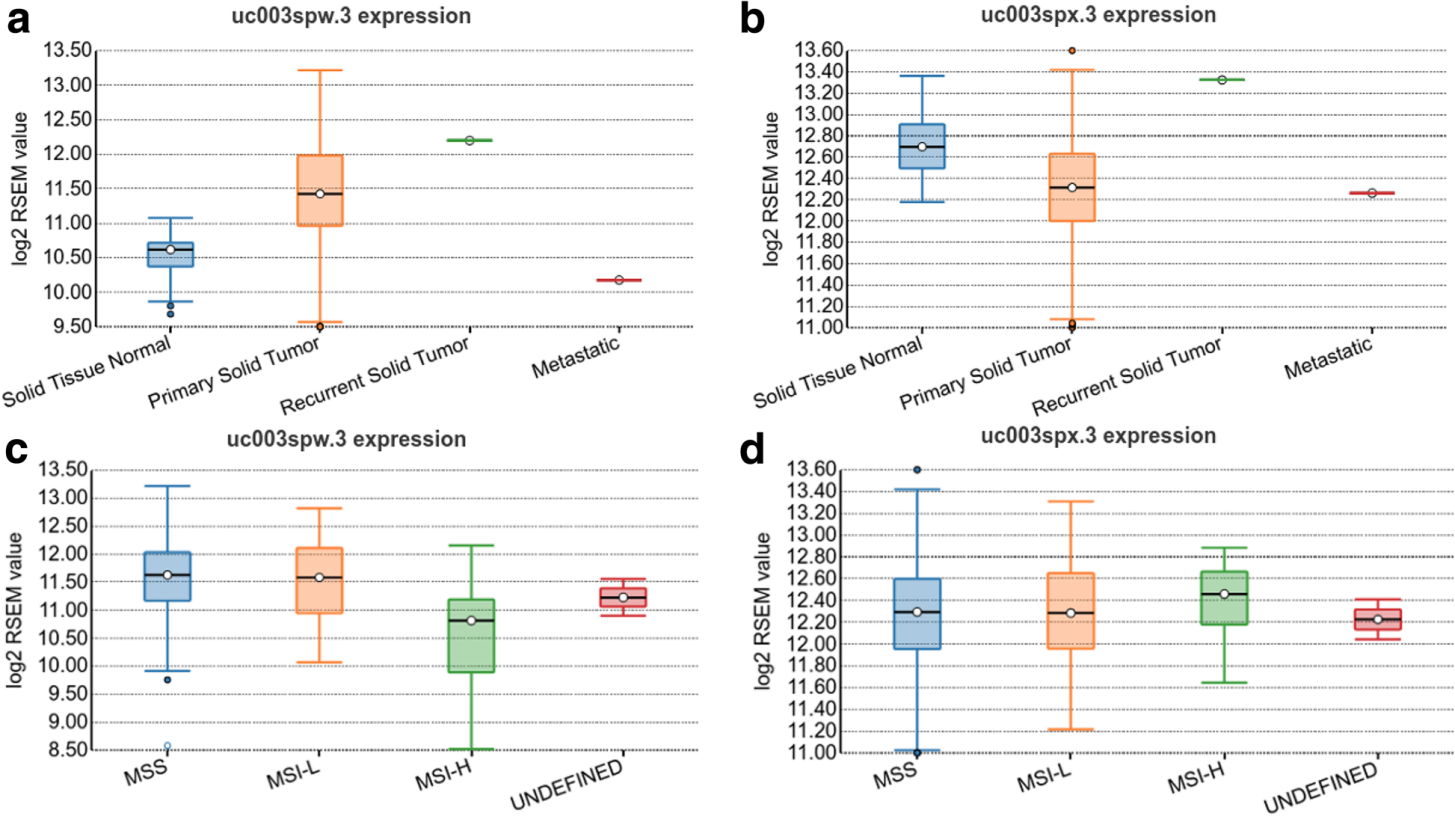
Expression of RAC1 splicing isoforms in colon adenocarcinoma in TSVdb. **a** and **b** Differential expression of isoforms uc003spw.3 and uc003spx.3, respectively, in tumor and normal tissues. **c** and **d** Differential expression of isoforms uc003spw.3 and uc003spx.3, respectively, in different MSI statuses

**Fig. 4:**
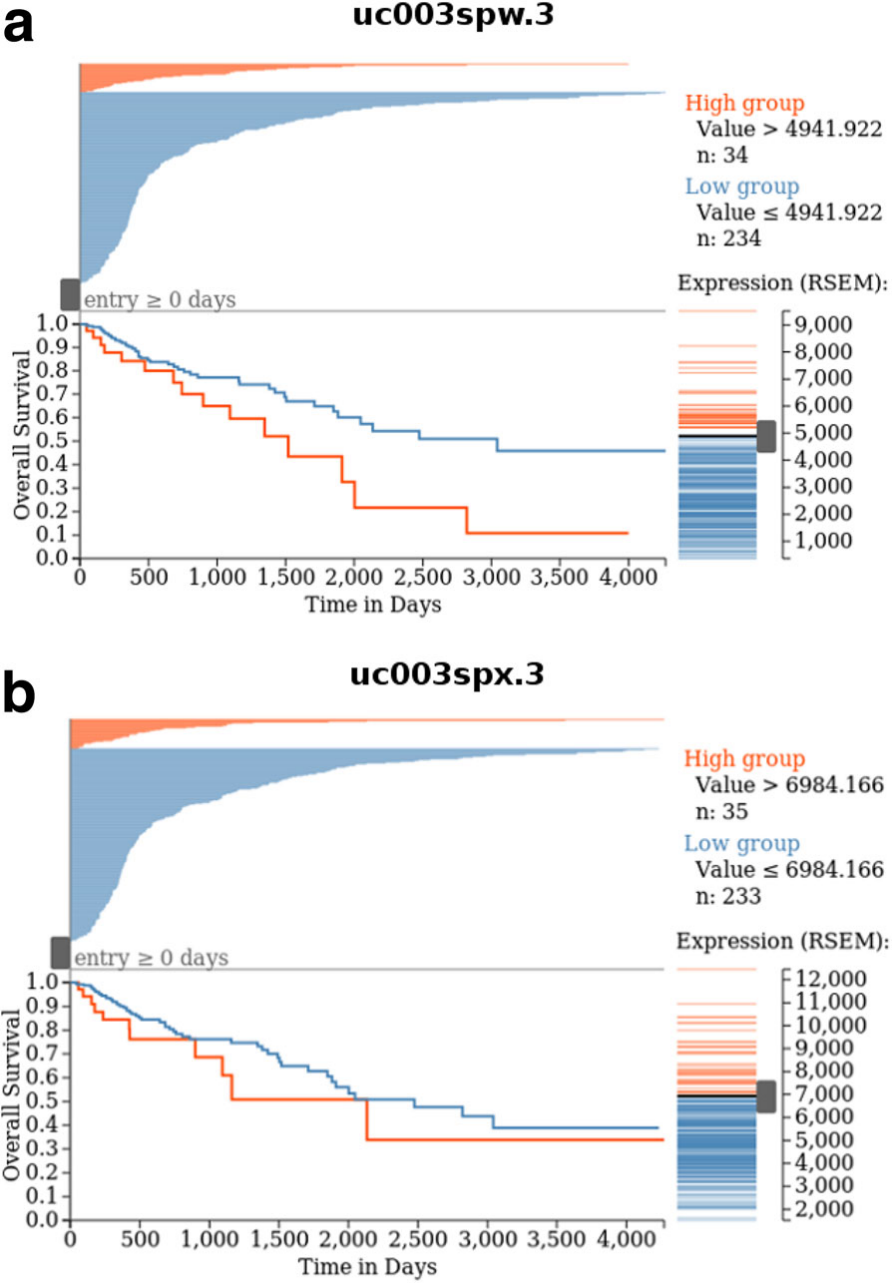
Kaplan-Meier plots showing the associations of the RAC1 isoform (**a**) uc003spw.3 and (**b**) uc003spx.3 with overall patient survival

Furthermore, the transcriptional pattern was also displayed to reveal the splicing isoforms for a single gene and their constitutions. Notably, by double-clicking the transcriptional pattern, the expression of the isoforms in the different subgroups was shown using a box plot (Fig. 3), and the correlation of the isoforms with overall survival was demonstrated by a KM-plot (Fig. 4), which indicated promising use for clinical cancer researchers. The KM survival results described four different parts as follows. (1) In the bottom right, there was a way to adjust the cut-off for grouping, where the knob could be adjusted to change the cut-off. The default value was set to the middle of the isoform expression range. (2) The top right part displayed information on the groups, including the cut-off value and sample size of each group. (3) The top left showed the survival line for each entered individual after filtering them by the survival start time. The start time could be changed by adjusting the knob. (4) The bottom left was the KM-plot. Moreover, users could also click on the right y-axis or bottom x-axis to invoke input boxes and set the position by inputting a specific value. The formatted exon/junction quantification, transcript isoform expression, clinical variables and gene expression data for a gene in one tumor type could be downloaded into one file by clicking the download link. An illustration of the downloaded table is shown in Additional file 1: Table S2

### Example analysis

The oncogene Rac Family Small GTPase 1 (RAC1) is closely associated with tumorigenesis, tumor progression and therapy resistance [14–16], and RAC1 alternative splicing is important for its regulatory role in cancers.[17]. As illustrated by RAC1 in colon cancer, RAC1 generates three splicing isoforms, and the fourth exon is skipped in normal tissues and included in tumor tissues (Fig. 2a). Consistently, it was revealed that the use of the junctions linking exons 3 to 4 and 4 to 5 were high in tumor tissues (Additional file 1: Figure S3A). Similarly, by choosing microsatellite instability (MSI) status as the phenotype, the results revealed that the fourth exon usage was high in the microsatellite stable (MSS) and microsatellite instability-low (MSI-L) groups (Fig. 2b), suggesting its potential role in DNA damage response [18]. Moreover, annotation from the UCSC Genome Browser (Fig. 2c) showed that the DNA sequence of the fourth exon was evolutionarily conserved in vertebrates, which indicated the importance of its function in tumor biology.

The isoform expression variation for RAC1 was also shown by the box plot (Fig. 3). Primary solid tumor tissues had higher uc003spw.3 transcript expression, while there was lower uc003spx.3 transcript expression relative to normal tissues (Fig. 3a and b) Interestingly, the fourth exon was only included in isoform uc003spw.3. Furthermore, the MSI-H samples showed lower uc003spw.3 transcript expression but higher uc003spx.3 transcript expression (Fig. 3c and d). Additionally, the KM-plots showed the correlation between uc003spw.3 and uc003spx.3 with the overall survival of colon cancer patients (Fig. 4), and the high uc003spw.3 expression was correlated with poor prognosis.

## Discussion

TSVdb is a user-friendly interface for unearthing alternative splicing variations in 33 cancers. Similar to existing tools, TSVdb provides a comparison of isoform expression and alternative splicing between tumor and normal samples, which can also be achieved by ISOexpresso [11] and TCGASpliceSeq [9], respectively (Table 1).

**Table 1:** Summary of the features of the three databases, including SpliceSeq, ISOexpresso, and TSVdb, for visualizing the TCGA splice variant data

Features	TCGA SpliceSeq	ISOexpresso	TSVdb
Splicing event measurements	Yes	No	No
Algorithm integrate exon, junction reads	Yes	No	No
Provide Screening result	Yes	No	No
Splicing pattern graph	Yes	No	No
Isoform pattern graph	No	Yes	Yes
Isoform expression	No	Yes	Yes
Exon quantification	Yes	No	Yes
Junction quantification	Yes	No	Yes
Clinical data	No	No	Yes
Multi-omics	No	Partial ^∗^	Partial ^*†*^
Genome-wide data download	Yes	No	No

^*^ISOexpresso provided eQTL analysis

^*†*^TSVdb provided gene expression correlated AS analysis

Moreover, TSVdb presents a better and more convenient visualization for users to assess exon/junction usage, transcript isoform expression, isoform pattern graph, and clinical information in one graph. This is the first time that we have integrated comprehensive clinical data with TCGA alternative splicing analysis tools; this web-tool will help to perform comparative analysis across different tumor subgroups. The subgroups are defined by demographic data, clinical diagnosis data, treatment data and follow-up data.

By taking advantage of clinical data from TCGA, in the analysis example, the result of the gene RAC1 was shown. We found that the expression of the fourth exon was high in adenocarcinoma tumor tissue. Moreover, it was discovered that exon usage mainly increased in tumor tissues from MSS and MSI-L patients.

In the data visualization tactic aspect, Sashimi Plots are most commonly used to visualize alternative splicing [19]. Sashimi Plots use read densities to represent the amount of reads alignment in exons or junctions. There are some variations of Sashimi Plots. For example, TCGASpliceSeq uses the number to show the reads quantity and GTEx project uses the color gradient to quantify. However, Sashimi Plots cannot visualize many samples as well and do not facilitate comparisons between samples. Visualizing Alternative Splicing (Vials) (http://vials.io/vials/) resolved the problem using a complex multivariable graph [20]. In TSVdb, the data visualization strategy was inspired by MEXPRESS [21], in which samples are plotted on the x-axis, the genome is arranged on the y-axis and comparisons between subgroups are achieved by sorting the samples by phenotypes. This strategy makes it possible to display data for hundreds of samples in a single figure with genome annotations. Although, as the price, TSVdb cannot display the exon and junction reads quantity simultaneously, it can take advantage of the big sample size in TCGA datasets.

## Conclusion

In summary, we provided a web-based tool for splicing variants analysis. We believe that TSVdb offers researchers a quick and straightforward visualization tool to explore alternative splicing and isoform expression of target genes in clinical subgroups within the TCGA data.

## Availability and requirements

**Project name:** TSVdb

**Project home page:** http://www.tsvdb.com/

**Operating system(s):** Platform independent

**Programming language:** R, Nodejs and perl 6 (server side scripts)

**Other requirements:** Internet browser required for network visualization

**License:** Creative Commons Attribution 4.0 International License (http://creativecommons.org/licenses/by/4.0/)

**Any restrictions to use by non-academics:** no restriction

## Additional files

Additional file 1 Supplementary figures and tables. **Table S1.** The clinical variable distribution. **Table S2**. The data download format. **Figure S1.** The query dialogs for choosing the tumor type. **Figure S2.** The procedures for calculating the RAC1 exon usage in colon adenocarcinoma. **Figure S3.** The RAC1 junction usage in colon adenocarcinoma using TSVdb. **Figure S4.** The Kaplan-Meier plots showing the associations of the RAC1 isoform uc003spw.3, uc003spx.3 with overall patient survival. (PDF 919 kb)

Additional file 2 MongoDB database scheme. (TXT 2 kb)

## Abbreviations

ACC: Adrenocortical carcinoma
AS: Alternative splicing
BLCA: Bladder urothelial carcinoma
BRCA: Breast invasive carcinoma
CESC: Cervical squamous cell carcinoma and endocervical adenocarcinoma
CHOL: Cholangiocarcinoma
COAD: Colon adenocarcinoma
DDR: DNA damage response
DLBC: Lymphoid neoplasm diffuse large B-cell lymphoma
ESCA: Esophageal carcinoma
GBM: Glioblastoma multiforme
HNSC: Head and neck squamous cell carcinoma
KICH: Kidney Chromophobe
KIRC: Kidney renal clear cell carcinoma
KIRP: Kidney renal papillary cell carcinoma
LAML: Acute myeloid leukemia
LGG: Brain lower grade glioma
LIHC: Liver hepatocellular carcinoma
LUAD: Lung adenocarcinoma
LUSC: Lung squamous cell carcinoma
MESO: Mesothelioma
MSI-H: microsatellite instability-high
MSI-L: microsatellite instability-low
MSS: microsatellite stable
OV: Ovarian serous cystadenocarcinoma
PAAD: Pancreatic adenocarcinoma
PCPG: Pheochromocytoma and Paraganglioma
PRAD: Prostate adenocarcinoma
RAC1: Rac family small GTPase 1
READ: Rectum adenocarcinoma
RSEM: RNA-Seq by expectation maximization
SARC: Sarcoma
SKCM: Skin cutaneous melanoma
SRSF1: Serine and arginine rich splicing factor 1
SRSF6: Serine and arginine rich splicing factor 6
STAD: Stomach adenocarcinoma
TCGA: The cancer genome atlas
TGCT: Testicular germ cell tumors
THCA: Thyroid carcinoma
THYM: Thymoma
UCEC: Uterine corpus endometrial carcinoma
UCS: Uterine carcinosarcoma
VM: Uveal melanoma
