# Supplementary material for: TSVdb: a web-tool for TCGA splicing variants analysis
Source: BMC Genomics. 2018 May 29;19:405. doi: 10.1186/s12864-018-4775-x (PMC5975414; doi:10.1186/s12864-018-4775-x)
Supplement: Supplementary file 1 — Supplementary figures and tables. Table S1. The clinical variable distribution. Table S2. The data download format. Figure S1. The query dialogs for choosing the tumor type. Figure S2. The procedures for calculating the RAC1 exon usage in colon adenocarcinoma. Figure S3. The RAC1 junction usage in colon adenocarcinoma using TSVdb. Figure S4. The Kaplan-Meier plots showing the associations of the RAC1 isoform uc003spw.3, uc003spx.3 with overall patient survival. (PDF 919 kb) [file 12864_2018_4775_MOESM1_ESM.pdf]

TSVdb: a web-tool for TCGA splicing variants analysis Supplymentary material

Contents

1 The clinical variable distribution 2

2 The data download format 3

3 The query dialogs for choosing the tumor type 4

4 The procedures for calculating the RAC1 exon usage in colon adenocarcinoma 5

5 The RAC1 junction usage in colon adenocarcinoma using TSVdb 6

6 The Kaplan-Meier plots showing the associations for the RAC1 isoform uc003spw 7

1 The clinical variable distribution

Table S1: The clinical variable distribution.

Varaible	Cancer type	n
gender	PAAD; GBM; COAD; UVM; ACC; READ; THYM; KIRC; DLBC; STAD; LUSC; LAML; KICH; KIRP; BLCA; PCPG; THCA; LUAD; ESCA; LGG; SKCM; MESO; CHOL; LIHC; HNSC; SARC; BRCA	27
race	PAAD; GBM; UCS; COAD; UVM; ACC; READ; OV; THYM; KIRC; DLBC; STAD; LUSC; LAML; CESC; KICH; KIRP; BLCA; PCPG; PRAD; THCA; LUAD; ESCA; LGG; SKCM; UCEC; MESO; BRCA; TGCT; CHOL; LIHC; HNSC; SARC	33
pr_receptor	BRCA	1
number_pack_years_smoked	LUSC; LUAD	2
location_in_lung	LUSC	1
laterality	ACC; TGCT; HNSC	3
h_pylori_infection	STAD	1
gleason_stage	PRAD	1
overall_survival	PAAD; GBM; UCS; COAD; UVM; ACC; READ; OV; THYM; KIRC; DLBC; STAD; LUSC; LAML; CESC; KICH; KIRP; BLCA; PCPG; PRAD; THCA; LUAD; ESCA; LGG; SKCM; UCEC; MESO; BRCA; TGCT; CHOL; LIHC; HNSC; SARC	33
masaoka_stage	THYM	1
family_history_of_cancer	PAAD	1
fab_morphology	LAML	1
er_receptor	BRCA	1
asbestos_exposure	MESO	1
tumor_tissue_site	THYM	1
pathological_stage	PAAD; COAD; UVM; ACC; READ; KIRC; STAD; LUSC; KICH; KIRP; BLCA; THCA; LUAD; ESCA; SKCM; MESO; BRCA; TGCT; CHOL; LIHC; HNSC	21
grade	CESC; UCEC; LIHC	3
clinical_stage	UCS; UVM; OV; DLBC; ESCA; UCEC; HNSC	7
tumor_site	SKCM	1
smoking_history	LUSC; CESC; ESCA	3
risk_factor	LIHC	1
reflux_history	STAD	1
microsatellite_instability	COAD; READ; STAD	3
history_of_colon_polyps	COAD; READ	2
her2_receptor	BRCA	1
alcohol_consumption	ESCA; HNSC	2
number_of_pregnancies	CESC; UCEC	2
menopause_status	CESC; UCEC	2
kras_mutation	LUSC; LUAD	2
histological_type	COAD; READ; THYM	3

2 The data download format

Table S2: The data download format.

Attribute	Example	Description
sampleID	"TCGA-3L-AA1B-01A"	TCGA sample id.
clinical_[clinical type]	"Primary Solid Tumor"	Clinical variables.
os_time	348	Overall survival time (day).
os_event	0	Overall survival event (0=live, 1=death).
gene_[gene name]	100.1234	Gene expression normalized read count.
isoform_[isoform id]	100.2345	Isoform expression normalized read count.
exon/junction_[location]	0.1102	Exon/junction usage value. Exon/junction usage is calculated by dividing exon/junction quantification value to mean exon/junction quantification value of that person for a specific gene

3 The query dialogs for choosing the tumor type

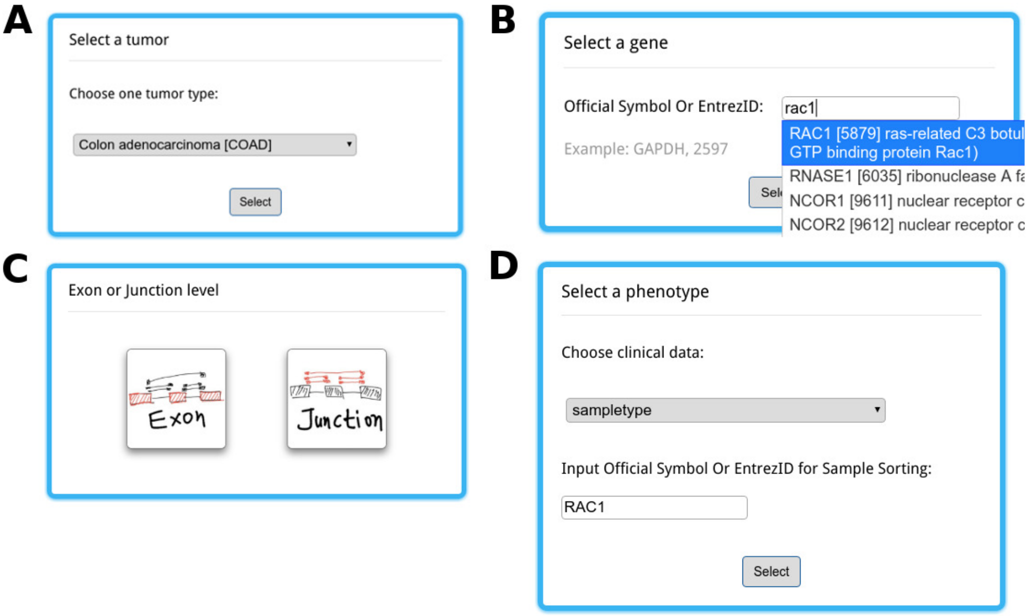

Figure S1: The query dialogs for choosing the tumor type (A), gene (B), exon/junction usage (C), phenotype and gene expression data that used to arrange samples in phenotype subgroups (D).

4 The procedures for calculating the RAC1 exon usage in colon adenocarcinoma

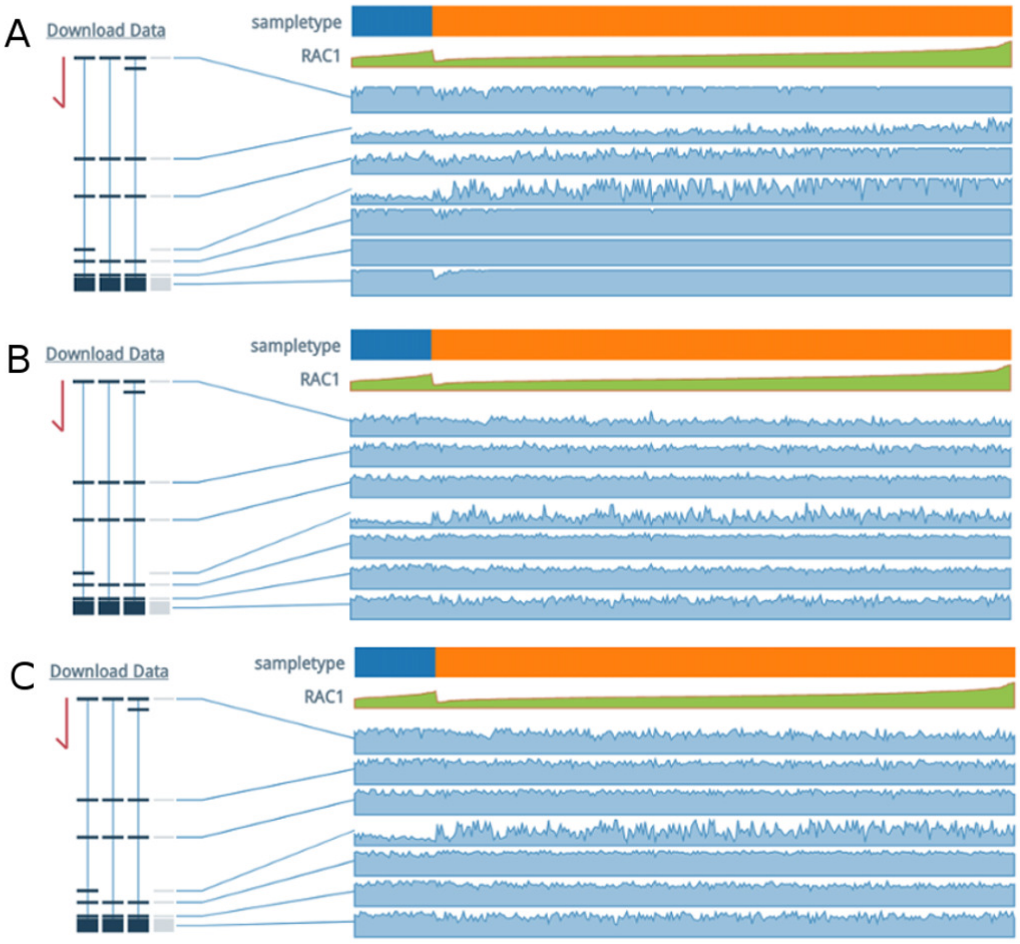

Figure S2: The procedures for calculating the RAC1 exon usage in colon adenocarcinoma. (A) Exon expression value (each peak represented for one sample value). (B) Exon usage value (the exon expression values were divided by gene expression). (C) The polished results of exon usage by diminishing the outlier effect.

5 The RAC1 junction usage in colon adenocarcinoma using TSVdb

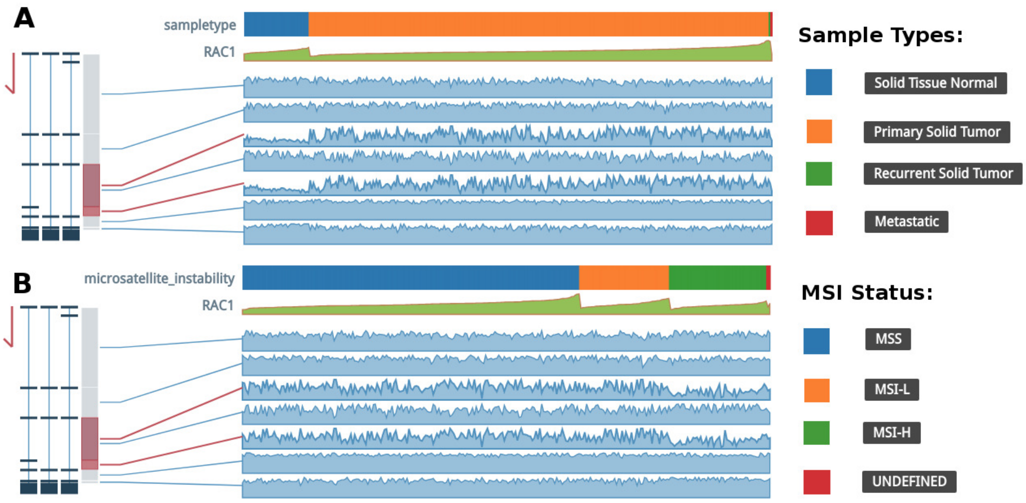

Figure S3: The RAC1 junction usage in colon adenocarcinoma using TSVdb. The different usage of junctions between sample types (A), and MSI status (B).

6 The Kaplan-Meier plots showing the associations for the RAC1 isoform uc003spw

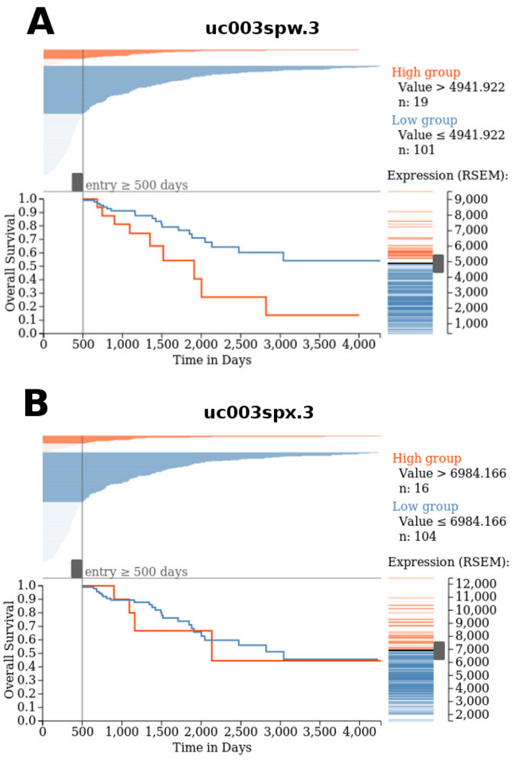

Figure S4: The Kaplan-Meier plots showing the associations for the RAC1 isoform uc003spw (A) and uc003spx.3(B) with patient overall survival after adjusting the onset.
